# Automated Morphological Analysis of Microglia After Stroke

**DOI:** 10.3389/fncel.2018.00106

**Published:** 2018-04-19

**Authors:** Steffanie Heindl, Benno Gesierich, Corinne Benakis, Gemma Llovera, Marco Duering, Arthur Liesz

**Affiliations:** ^1^Institute for Stroke and Dementia Research, University Hospital, Ludwig-Maximilians-Universität München, Munich, Germany; ^2^Munich Cluster for Systems Neurology (SyNergy), Munich, Germany

**Keywords:** microglia, morphology, stroke, image analysis, neuroinflammation

## Abstract

Microglia are the resident immune cells of the brain and react quickly to changes in their environment with transcriptional regulation and morphological changes. Brain tissue injury such as ischemic stroke induces a local inflammatory response encompassing microglial activation. The change in activation status of a microglia is reflected in its gradual morphological transformation from a highly ramified into a less ramified or amoeboid cell shape. For this reason, the morphological changes of microglia are widely utilized to quantify microglial activation and studying their involvement in virtually all brain diseases. However, the currently available methods, which are mainly based on manual rating of immunofluorescent microscopic images, are often inaccurate, rater biased, and highly time consuming. To address these issues, we created a fully automated image analysis tool, which enables the analysis of microglia morphology from a confocal Z-stack and providing up to 59 morphological features. We developed the algorithm on an exploratory dataset of microglial cells from a stroke mouse model and validated the findings on an independent data set. In both datasets, we could demonstrate the ability of the algorithm to sensitively discriminate between the microglia morphology in the peri-infarct and the contralateral, unaffected cortex. Dimensionality reduction by principal component analysis allowed to generate a highly sensitive compound score for microglial shape analysis. Finally, we tested for concordance of results between the novel automated analysis tool and the conventional manual analysis and found a high degree of correlation. In conclusion, our novel method for the fully automatized analysis of microglia morphology shows excellent accuracy and time efficacy compared to traditional analysis methods. This tool, which we make openly available, could find application to study microglia morphology using fluorescence imaging in a wide range of brain disease models.

## Introduction

Microglia are the resident immune cells in the brain and are essential for the phagocytosis of pathogens and apoptotic cells as well as for modulating adaptive immune responses (Aloisi, 2001; Prinz and Priller, 2014; Colonna and Butovsky, 2017; Wolf et al., 2017). Under physiological conditions, mature microglial cells are highly ramified with a small soma and very fine, long processes, which allow them to screen the brain parenchyma for invading pathogens or damage associated molecular patterns (DAMPs) (Colonna and Butovsky, 2017). In this state, microglia were previously described as “resting.” However, more recent studies demonstrate them to be highly dynamic and should rather be perceived as “surveilling” microglia (Tremblay et al., 2011). DAMPs in the microglial environment initiate a rapid morphological transformation of microglia from a ramified into an amoeboid cell type, which allows them to migrate to the site of injury or to phagocytose (Nimmerjahn et al., 2005; Tremblay et al., 2011). Tissue injury to the central nervous system (CNS), such as during an ischemic stroke, leads to the massive release of DAMPs, reactive oxygen species (ROS), and other inflammatory mediators. Further, blood-borne immune cells can infiltrate into the ischemic brain, promote post-ischemic inflammation and increase the release of pro-inflammatory cytokines (Gelderblom et al., 2009; Tremblay et al., 2011; Prinz and Priller, 2017). Consequently, surveilling microglia react to the inflammatory milieu and become “activated” within minutes after ischemia onset, which is characterized by an increased release of pro- or anti-inflammatory cytokines, a change in surface molecule expression, and a morphological transformation from ramified into amoeboid microglia (Kriz, 2006; Kettenmann et al., 2011; Tremblay et al., 2011; Kawabori and Yenari, 2015; Wolf et al., 2017).

In contrast to the common perception, the morphology of microglia is not strictly limited to two classes of either ramified or amoeboid shape but can present the whole range of morphological changes and also subtle changes (Stence et al., 2001; Fumagalli et al., 2013). Morphological variety is especially known for microglia in the ischemic brain, where different morphologies were found at varying distance from the ischemic lesion, showing that microglia in the proximity of the lesion are amoeboid, whereas microglia in the contralateral hemisphere distant to the lesion preserve the highly ramified shape (Fumagalli et al., 2013; Butovsky et al., 2014; Anttila et al., 2016; Krasemann et al., 2017). Hence, quantifying microglia morphology has been used in numerous studies as a key marker for microglial activation and function after stroke (Faustino et al., 2011; Perego et al., 2011; Morrison and Filosa, 2013; Anttila et al., 2016). To date, there is only a limited set of tools to properly quantify microglia morphology. Commonly, these methods are based on immunohistology of brain slices using markers such as CX3CR1 (fractalkine receptor), CD11b, and Iba1 (Perego et al., 2011; Morrison and Filosa, 2013; Wolf et al., 2013). Extraction of morphological information is usually achieved by manual procedures such as particle analysis and Sholl analysis (Derecki et al., 2014; Xu et al., 2016), which are based on two-dimensional projections of microscopic images (Perego et al., 2013; Derecki et al., 2014). The currently available methods for evaluating microglia morphology have several limitations to usability, sensitivity and robustness. Analysis of two-dimensional projections leads to substantial loss of information when analyzing complex three-dimensional cell shapes. On the other hand, manually performed methods are prone to rater bias and limited comparability of results between raters. Finally, a key limitation of manual analysis procedures is their high time demand, which renders these methods less efficient and not applicable for analysis of large cell numbers.

To address all the limitations of manual microglial shape analysis, we developed a fully automated algorithm, which quantifies microglia morphology from confocal Z-stacks in a fast and unbiased manner. Based on this algorithm, we can provide a broad panel of shape descriptors, which are suitable for characterization of microglia morphology. After developing the algorithm on an exploratory dataset, we validated its performance on an independent dataset and assessed comparability to currently prevailing manual analyses.

## Materials and methods

### Animals

All experiments in this study were conducted in accordance with the national guidelines for animal experiments and approved by the German governmental committees (Regierung von Oberbayern, Munich, Germany). The animals were 10–12 weeks old, male C57BL/6J mice (25–23 g body weight, Charles River Laboratories), housed under controlled temperature (22 ± 2°C) with a 12-h light-dark cycle and access to food and water *ad libitum*. Post-surgery analgesia and sedation protocols were conducted in accordance with approved protocols by the governmental committee.

### Experimental paradigm

**Figure 1:**
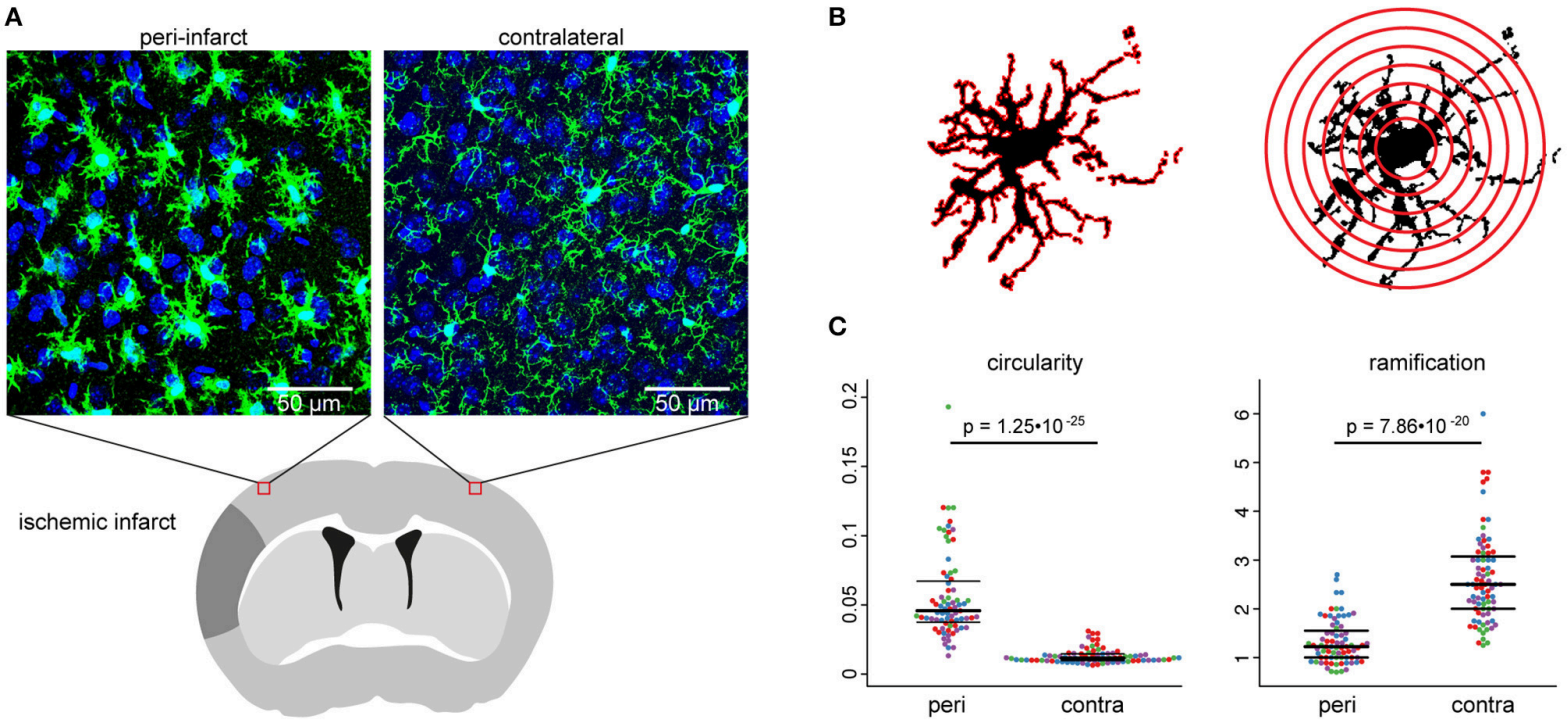
Experimental paradigm. The stroke model **(A)**, used for the development of the automated algorithm, was first analyzed manually **(B)**, to evaluate the impact of the infarct on microglia morphology **(C)**. **(A)** The scheme depicts the regions of interest for confocal imaging in the peri-infarct area and the homotypic area in the contralateral cortex 3 days after stroke. Images show representative maximum intensity projections of image stacks at both positions from Iba1-stained brain sections. **(B)** The schemes illustrate graphically the calculation base for the circularity index (left) and Shoenen ramification index (right) for the manual analysis. **(C)** Swarm plots show individual cells in the peri-infarct area (peri) and in the contralateral hemisphere (contra) for the manually analyzed circularity index (left) and Shoenen ramification index (right). Seventy-five to seventy-nine cells per region of *n* = 4 mice, colors correspond to individual mice.

For the development of the automated algorithm for the morphological analysis of microglia, we used a stroke model. Microglia activation and consecutive morphological changes after an ischemic infarct were used to establish features sensitive to different morphologies. Confocal image stacks were acquired from two locations in the cortex in cortical layer II/III (see Figure 1A). The first location was 300 μm away from the infarct (peri-infarct area), where microglia activation was to be expected high. The second location was in the homotypic contralateral cortex (contra), where activation is expected to be less strong or even absent. These images were acquired from nine mice, which were all treated in an identical manner. The images were then split randomly into two datasets. An exploratory dataset contained the images of four mice and was used for the development of the automated algorithm (hereafter stated as “exploratory” dataset). A validation dataset contained the images of the other five mice and was used for validation of the performance of the algorithm (hereafter stated as “validation” dataset).

For quantification of subtle morphological differences between activated cells in the peri-infarct area, an additional experiment was conducted with five further mice. In this experiment, multiple image stacks were acquired in a grid-like spatial arrangement and analyzed by the automated algorithm.

### Distal middle cerebral artery occlusion

For anesthetic induction, mice were deeply sedated with 4% isoflurane until full loss of motor responses. During the surgery, mice were kept under inhalation anesthesia with a mixture of isoflurane, nitrous oxide, and oxygen (2/70/30) provided with an inhalation mask and at a body temperature of 37°C using a heating pad, monitored with a rectal thermometer. For pre-medication, mice were treated with 0.1 mg/kg bodyweight Buprenorphine by subcutaneous injection. Ischemia was induced by applying the distal middle cerebral artery occlusion (dMCAO) model of transcranial and permanent coagulation of the middle cerebral artery (MCA) via electrocoagulation distal to the lenticulostriatal arteries as previously described (Llovera et al., 2014). In brief, a 1 cm long incision was made between the left eye and the ear and the temporal muscle was removed from the skull with electrocoagulation forceps. After identifying the MCA below the transparent skull, the bone covering the MCA bifurcation was thinned and removed. The artery was permanently coagulated proximal and distal to the MCA bifurcation. After the surgery, the animal was placed in a nursing box at 32°C for 10 min to recover from the anesthesia and treated with Carprofen 4 mg/kg bodyweight intraperitoneally until the third day after the surgery. Since the main goal of this study was to investigate microglia morphology by an automated algorithm, but not to test effects of therapeutic approaches, the use of Carprofen was not seen as an obstacle.

### Cardiac perfusion

The animals were anesthetized at day 3 (exploratory and validation study) or day 5 after stroke (peri-infarct morphology study) with a lethal dose of Ketamine (120 mg/kg) and Xylazine (4 mg/kg). The abdomen and thorax were opened, and an incision was made into the right atrium. Mice were perfused with 20 ml of cold isotonic 0.9% saline via the left ventricle and subsequently perfused with 15 ml ice-cold 4% paraformaldehyde (PFA) in PBS.

### Tissue preparation and immunofluorescence staining

After PFA-perfusion, the brains were rapidly removed and post-fixed in 4% PFA for 18 h and dehydrated in 30% sucrose for at least 48 h until the brains were completely sunk to the bottom of a 15 ml Falcon tube. Then, 100 μm coronal sections were cut using a Leica Vibratome and collected in 0.1 M phosphate-buffered saline (PBS). Staining was performed with free floating sections in 24-well plates. The sections were blocked with goat serum blocking buffer (2% goat serum, 1% bovine serum albumin, 0.1% gelatin from cold water fish skin, 0.1% Triton X-100, 0.05% Tween 20 in 0.01 M PBS, pH 7.2–7.4) in 24-well plates and stained with 1:200 anti-Iba1 (rabbit, Wako, #019-19741) in primary antibody buffer (1% bovine serum albumin, 0.1% gelatin from cold water fish skin, 0.5% Triton X-100 in 0.01 M PBS, pH 7.2–7.4) and anti-rabbit coupled to Alexa-fluor 594 (goat anti-rabbit, Thermo Fisher Scientific, #A-11012) in secondary antibody buffer (0.05% Tween 20 in 0.01 M PBS, pH 7.2–7.4). Nuclei were stained with 4′,6-Diamidin-2-phenylindol (DAPI, Invitrogen, #D1306) 1:5,000 in 0.01 M PBS. Sections were mounted on microscope slides (Menzel-Gläser Superfrost® Plus, Thermo Fisher Scientific, #3502076) and covered with a coverslip (Menzel-Gläser 24–60 mm, #1, BB024060A1, Wagner und Munz) using aqueous mounting medium (Fluoromount^TM^, Sigma-Aldrich, #F4680-25ML).

### Image acquisition

Images were acquired using a Zeiss confocal microscope with 40x magnification (objective: EC Plan-Neofluar 40x/1.30 Oil DIC M27) with an image matrix of 1024 × 1024 pixel, a pixel scaling of 0.2 × 0.2 μm and a depth of 8 bit. Confocal-images were collected in Z-stacks with a slice-distance of 0.4 μm.

### Manual analysis of microglia morphology

Manual morphological analysis was performed using FIJI software (Version 2.0) and the Sholl analysis plugin (Ferreira et al., 2014). Z-stacks were imported into the software and the slices containing individual Iba1 positive cells were identified by manually scrolling through the Z-stack. Only slices containing an identified cell were processed as a maximum intensity projection to avoid overlapping with cells located in more superficial or deeper layers. Maximum intensity projections of the cells were thresholded for creating a binary mask. By measurement of the area (black) and the perimeter (red) of a cell from the binary mask, the circularity index (CI) was calculated (4π[area]/[perimeter]^2^) (Figure 1B, left). For Sholl analysis, the maximum radius of the cell soma and the radius surpassing the longest branch of the cell were measured and the number of primary branches was manually counted (Figure 1B, right). Further, default settings of the FIJI Sholl analysis plugin were used [Radius step size = automatic (“0.00”), Enclosing radius cutoff = 1 intersection, Sholl Method = linear] and the Shoenen ramification index (RI) was calculated (number of end branches/number of primary branches) and collected for each cell. For each animal, a minimum of 15 cells was analyzed per imaging region (between 15 and 24 cells depending on the number of detectable cells in a Z-stack due to variable cell numbers in the analyzed locations).

### Automated analysis

**Figure 2:**
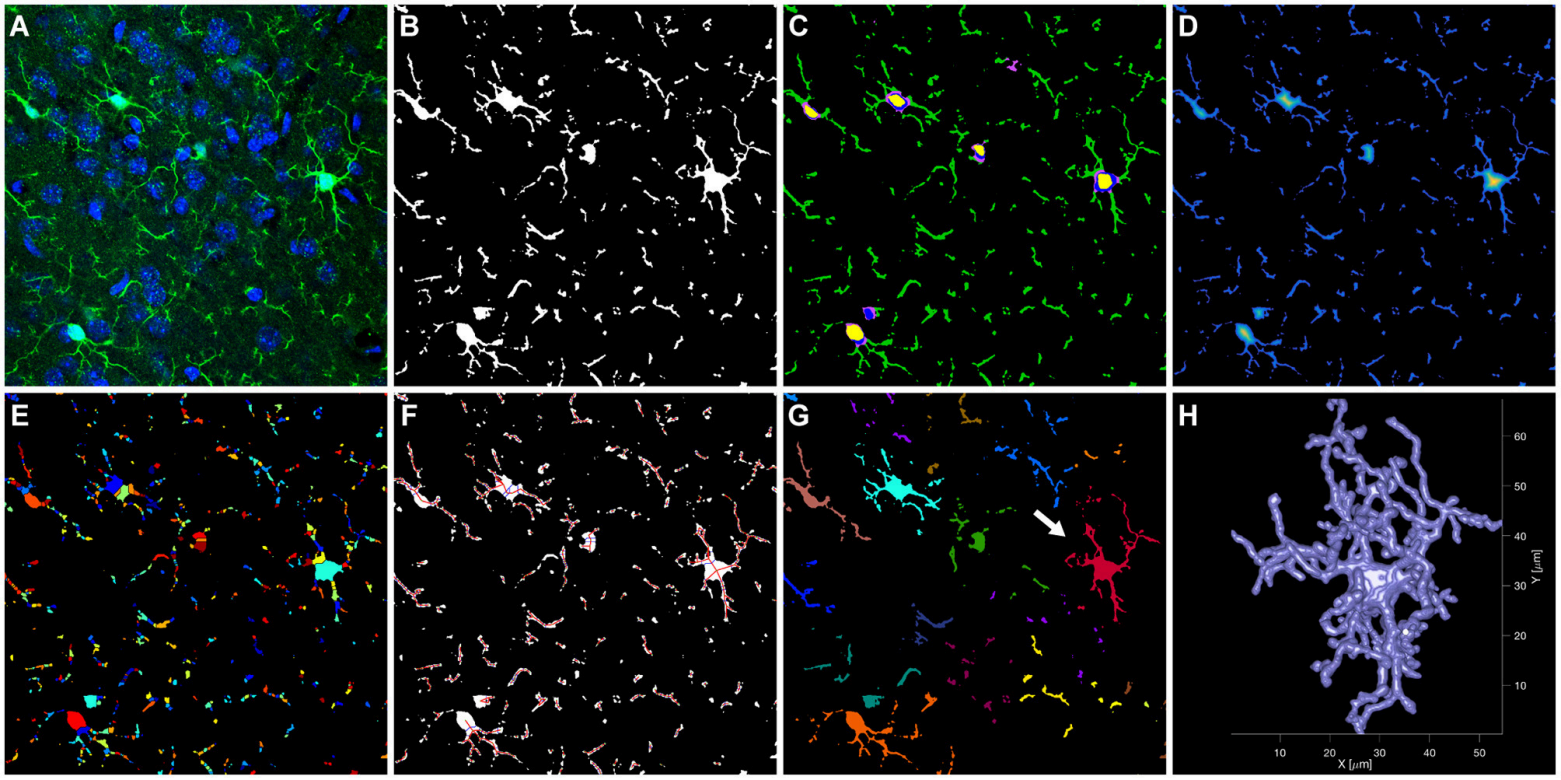
Main steps of the automated analysis of microglial cells. The raw confocal image (**A**, shown as single slice of the Z stack) was segmented into microglial cells and background **(B)**. Cells were further segmented **(C)** into nucleus (yellow), soma (blue/purple), and branches (green). Subsequently, a skeleton was created in three steps. A distance map was calculated, indicating for each voxel the distance to the cell surface (D, distance increases from blue to yellow). This distance map was then used as input to a watershed segmentation (**E**, colors were chosen at random for each segment). The resulting segments were connected to create the skeleton **(F)**. For visualization, a simplified skeleton in 2D is shown. The skeleton was then used to segregate individual cells (**G**, each color represents a different cell). The surface model of the red cell in **(G)** (arrow) is shown in **(H)**.

The fully automated processing of confocal image stacks and the extraction of morphological features consisted of four main steps: (i) Control of image quality and preprocessing (ii) segmentation of microglial cells from background, and segmentation of cells into nucleus, soma, and branches. (iii) Construction of a skeleton to represent the spatial structure of cell bodies and branches. (iv) Extraction of morphological features using properties derived from the surface area, volume, and skeleton of the cells. These main steps and the key intermediary steps are illustrated in Figure 2 and are described in more detail below. The whole processing pipeline was implemented in MATLAB (R2016b, The MathWorks, Natick, Massachusetts, USA), using custom written scripts, with dependencies on the Image Processing Toolbox and Statistics and Machine Learning Toolbox. The processing of a single confocal image stack lasted on average 80 min on a computer with a modern CPU (Intel Core i7) and 16 GB RAM. Note however, that processing of multiple stacks can easily be parallelized and that the pivotal criterion for comparing manual and automated analysis is the hands-on time needed to analyze the image stacks (see section Results). The MATLAB scripts can be downloaded at https://github.com/isdneuroimaging/mmqt.

#### Quality control and pre-processing

Raw image Z-stacks (Zeiss CZI files) were read into MATLAB using the Bio-Formats software tool for MATLAB (Open Microscopy Environment consortium) (Linkert et al., 2010). In order to evaluate image quality and remove low quality images from the stack, spatial correlations between neighboring images in the stack were calculated. This procedure follows the rationale that the properties of the tissue do not change completely within the 0.4 μm distance between images, but show high similarities. However, if the signal to noise ratio in the image decreases, these similarities will decrease, which can be assessed by a decline in spatial correlation between images (see Supplementary Figure 1). We removed images from the top and bottom of the Z-stack, whose spatial correlations with the neighboring image yielded a coefficient below 0.78. This threshold was determined in the exploratory data set as being most similar to a human rater performing visual inspection. While a deconvolution step might be useful under specific circumstances (e.g., oversampling at very high Z resolution) it did not improve image quality in our data set.

#### Image segmentation

Raw images (Figure 2A) were smoothed using a Gaussian kernel with a standard deviation of 0.3 μm. Microglia whole cells and nuclei were then segmented from the background (see Figures 2B,C) using thresholds determined individually for each image in the Z-stack and each channel (i.e., anti-Iba1 and DAPI staining), based on the intensities at the edges found in the images. For details on selecting these thresholds and creating binary masks for microglial cells and nuclei see Supplementary Methods. The resulting 3D masks were refined by removing clusters of voxels with very small volume, and by filling holes and gaps in the masks. Additional masks for branches, soma and nuclei of the microglial cells were defined during and after this refinement procedure. For details see Supplementary Methods.

#### Definition of the skeleton and segregation of cells

A skeleton was used to capture the main structural components of the microglial cells and their configuration. This skeleton was created in three steps (see Figures 2D–F). A distance map was calculated based on the binary mask of the microglial cells, indicating for each voxel inside a cell the shortest distance to the cell surface. This distance map was then broken up into segments using a watershed segmentation. In a third step, the centroids of the resulting segments were connected by straight lines, in order to create the skeleton. This skeleton can also be defined and analyzed mathematically as a graph consisting of nodes and edges, with the centroids of the watershed segments corresponding to the nodes, and the connecting lines corresponding to the edges. For details on refinement of the skeleton and definition of major cell branches and cycles in the branches, see Supplementary Methods. Supplementary Video 1 shows an example Z-stack and its skeleton. After the skeleton was defined for the entire Z-stack, connected microglial cells were segregated on the basis of the skeleton properties (Figure 2G). To this end, one microglial cell was defined for each cell soma, by taking for each soma the skeleton node at its center and calculating the shortest path between these nodes and all other nodes in the entire stack. Each node was then assigned to a soma by determining the minimum for the shortest path. A 3D representation of an individual cell after segregation (highlighted in Figure 2G) is shown in Figure 2H and Supplementary Video 2. To ensure that only cells with visible major branches were included in the analysis, we excluded cells when parts of them touched a border of the Z-stack and the centroid of the soma was closer to that border than 15 μm (X/Y borders) or 8 μm (Z borders), respectively. In addition, cells were excluded if they were connected to each other and the centroid of their soma were closer together than 15 μm. This second rule was used to remove cells from the analysis for which the separation into single cells was not feasible.

#### Definition of morphological features

We defined 59 features, describing the shape and spatial structure of each individual microglial cell. The features were selected from the three domains: (1) basic shape descriptors (based on volume, surface area, perimeter), (2) skeleton properties in order to account for the specific properties of microglia as ramified cells and (3) graph theory using established graph theoretical parameters (Supplementary Table 1, for review about graph theory see Rubinov and Sporns, 2010). For features which could be defined for each node or for each branch of a skeleton, the scores of 5 percentiles were extracted: minimum, 25th percentile, median, 75th percentile, and maximum.

#### Comparison of automated and manual analysis

Circularity was calculated in the exact same way in the manual and automated analysis. Slightly different procedures had to be used for features assessing ramification. While Shoenen ramification index was used in the manual analysis, the comparable feature “end-nodes per branch” was obtained by the automated analysis.

### Statistical analysis

All statistical analyses and data visualizations were performed in RStudio (RStudio Team, 2016) using R version 3.2.2 (R Core Team, 2016) and the packages ROCR (Sing et al., 2005), plyr (Wickham, 2011), beeswarm (Eklund, 2016), and corrplot (Wei, 2016). A Wilcoxon rank sum test with continuity correction was applied for group comparisons unless otherwise specified in the respective Results section.

To identify the best features capturing morphology changes after microglia activation, we evaluated the ability to discriminate between cells in the peri-infarct area and in the contralateral hemisphere by receiver operating characteristic (ROC) analysis. The area under the curve (AUC) was used as a measure of discrimination performance.

To reduce the dimensionality of the feature set, we performed a principle component analysis (PCA) with centering and scaling of the features. Only features which had an AUC score above 0.85 were included in the PCA. If a feature (e.g., volume of the skeleton nodes) was determined with different percentiles (e.g., median over all nodes, or 75th percentile, see above), we included only the variant with the highest AUC score in the PCA.

## Results

### Manual analysis of microglia morphology detects differences between peri-infarct and contralateral brain areas

Manual analysis of microglial cells in the exploratory dataset showed significant differences between cells in the two studied regions (i.e., peri-infarct area and homotypic contralateral cortex; see Figure 1A), for both extracted features (Figures 1B,C). Microglia in the peri-infarct area were more circular than microglia in the contralateral cortex, whereas the ramification index revealed that microglia in the contralateral cortex were more ramified than in the peri-infarct area. This result ensured the exploratory dataset to be appropriate for designing an automated algorithm, which shall quantify different microglia morphologies.

### Establishing an automated analysis tool for microglial shape analysis

**Figure 3:**
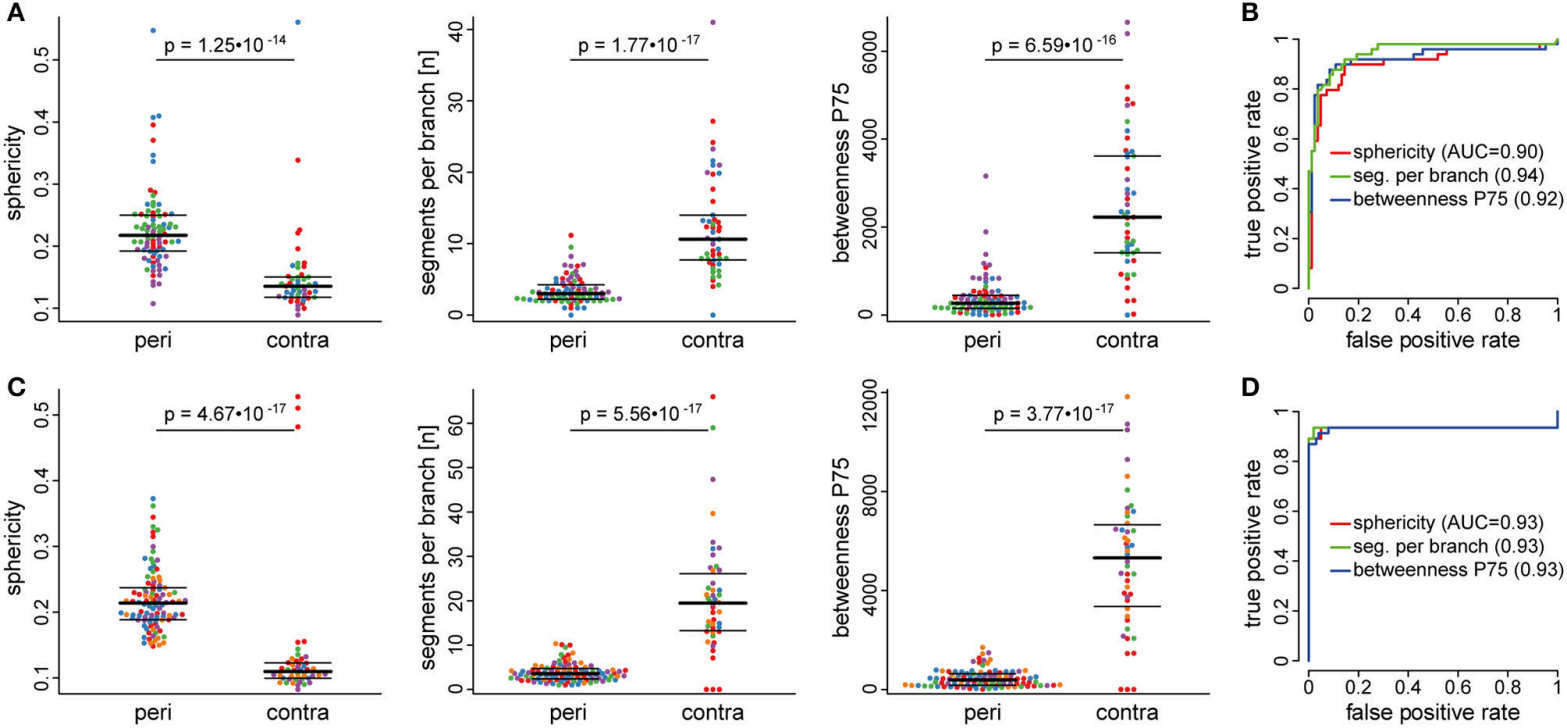
Automatically extracted morphological features in peri-infarct and contralateral cortex. Automated analysis of microglia morphology was performed in an exploratory dataset **(A,B)** and an independent validation cohort **(C,D)**. Swarm plots in **(A,C)** show individual cells in the peri-infarct area (peri) and in the contralateral hemisphere (contra) for three representative features: sphericity, segments per branch, and betweenness. Colors in the swarm plots correspond to individual mice. The receiver operating characteristic (ROC) curves indicate very good discrimination performance for all three features, both in the exploratory **(B)** and the validation **(D)** dataset.

We aimed to develop an algorithm for fully automated analysis of microglial cells using the same exploratory dataset as for the manual analysis shown above. Our algorithm used 59 shape features to describe microglia morphology. These features can be divided into three different types, depending on the underlying calculation: 12 features were based on simple shape properties (e.g., sphericity), 29 features were based on skeleton properties (e.g., number of segments per branch), and 18 features were based on graph theoretical measures (e.g., betweenness). One representative feature from each category was selected for illustration (Figures 3A,B).

The performance of all 59 features to discriminate microglia morphology between the peri-infarct and the contralateral cortex, as assessed by ROC analysis, is presented in Supplementary Table 1. ROC curves illustrate the discrimination performance of a binary classifier system by plotting the true positive rate against the false positive rate at various thresholds. Discrimination performance is quantified by the area under the curve (AUC), ranging from 0.5 (chance level) to 1 (perfect discrimination). In our analyses, seventeen features showed very high AUC values (AUC > 0.85), indicating an excellent performance to discriminate between cell morphology in the peri-infarct and the contralateral area (Table 1).

**Table 1:** Morphological features with high discrimination performance (AUC > 0.85).

**Label**	**AUC**	Type^*^	**Definition**
Sphericity	0.90	Simple	Measure of compactness of the cell in 3D
Circularity	0.91	Simple	Measure of compactness of the cell in 2D
Volume P75	0.93	Simple	Volume of nodes; 75th percentile
Nodes total	0.89	Skeleton	Total number of nodes in the skeleton
Branching nodes	0.90	Skeleton	Number of branching nodes (degree > 2)
End-nodes	0.86	Skeleton	Number of blind ending nodes (degree = 1)
Nodes in branches	0.90	Skeleton	Number of nodes located in the branches
Nodes per branch	0.94	Skeleton	Nodes per major branch
End-nodes in branches	0.89	Skeleton	Number of nodes located in the branches, having degree 1 (i.e., branch end-nodes)
End-nodes per branch	0.91	Skeleton	End-nodes per major branch
Branch segments	0.91	Skeleton	Number of branch segments
Segments per branch	0.94	Skeleton	Segments per major branch
Branch cycles	0.91	Skeleton	Number of cycles in the branches
Branch length skeleton P75	0.90	Skeleton	Length of major branches, following the edges of the skeleton; 75th percentile
Branch sinuosity P50	0.90	Skeleton	Ratio branch length skeleton/branch length air-line; median
Closeness P75	0.92	Graph	Closeness of nodes; 75th percentile
Betweenness P75	0.92	Graph	Betweenness of nodes; 75th percentile

* *Indicates whether the feature is based on simple shape properties (simple), skeleton properties (skeleton) or graph theoretical measures of centrality (graph). For detailed information about the features, see Supplementary Table 1*.

To validate our algorithm, we analyzed an additional, independent dataset of five mice. Tissue processing and imaging location were identical to the exploratory cohort and the same 59 features were extracted. Discrimination performance in the validation dataset was similar compared to the exploratory dataset, as indicated by a high correlation between AUC values from the exploratory and validation dataset (Pearson's *r* = 0.93, Supplementary Figure 2). For comparison with the exploratory dataset, the same representative features are visualized for the validation dataset in Figures 3C,D.

### Dimensionality reduction allows to analyze microglia morphology by a compound score

**Figure 4:**
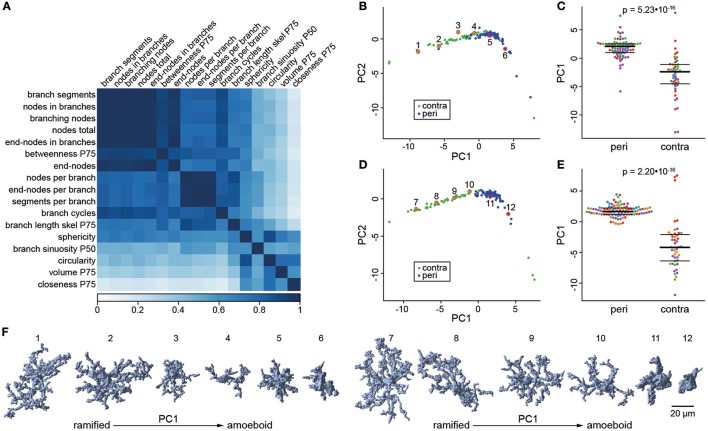
Dimensionality reduction by principal component analysis. The correlation matrix **(A)** was calculated for the 17 selected features with an AUC > 0.85 in the ROC analysis. This matrix indicates a high degree of multicollinearity among features and hence suggests that principle component analysis (PCA) is suited to reduce the dimensionality of this feature set. **(B)** Scores for the first and second principle component (PC1/PC2) of cells in the exploratory dataset. Cells are colored according to their location in the contralateral hemisphere (contra; green) or the peri-infarct area (peri; blue). **(C)** Swarm plots for PC1, with the same plotting conventions as used in Figure 3. **(D,E)** The same plots for the validation dataset as shown in panel **(B,C)**. In both datasets, PC1 discriminates very well between cells from peri-infarct and contralateral cortex. **(F)** Three-dimensional (3D) representation of microglial cells along PC1 in the exploratory (cells 1–6) and validation (cells 7–12) dataset. The plotted cells are indicated by numbered red circles in panel **(B,D)**.

Next, we aimed to reduce the dimensionality of the whole feature set. A correlation matrix indicated a high degree of multicollinearity among the 17 features with AUC values above 0.85 in the exploratory dataset (Figure 4A). Therefore, we performed a principle component analysis (PCA) to create a morphology compound score (Table 1).

In the exploratory dataset, the first two principal components explained 85% of the variance in the feature set (Figure 4B). The first PC (explaining 71% of variance alone) distinguished very well between the morphology of microglia in the peri-infarct area and in the contralateral cortex (Figure 4C). Accordingly, the discrimination performance was very high with an AUC score of 0.92. PCA in the independent validation dataset confirmed these results. The first principal component (explaining 73% variance) showed a comparable difference between the two locations and again a very good discrimination performance (Figures 4D,E, AUC = 0.93). Taken together, the first principle component provides a compound score with high discrimination performance, which has the advantage of being based on multiple shape features, and thus higher robustness than any single feature. Importantly, there was no indication for distinct morphology classes, but a continuum of gradual changes along PC1. This is further illustrated by six representative cells plotted along PC1 in both the exploratory and the validation dataset (Figure 4F). PC1 robustly quantified the degree of ramification along the two extremes, the ramified/surveilling and the amoeboid/activated microglial phenotype.

The PCA also revealed four outlier data points from microglial cells in the contralateral control area (Supplementary Figure 3). We evaluated these cells using the backtracing functionality of our algorithm and have determined that these cells were erroneously identified by the algorithm as cells although they represent only nuclei of other cell populations. The error rate for false-positive cell identification is thereby approximately 1% (4 out of 417 total cells in the exploratory and validation data set).

### The automated analysis detects subtle morphological differences between activated cells

**Figure 5:**
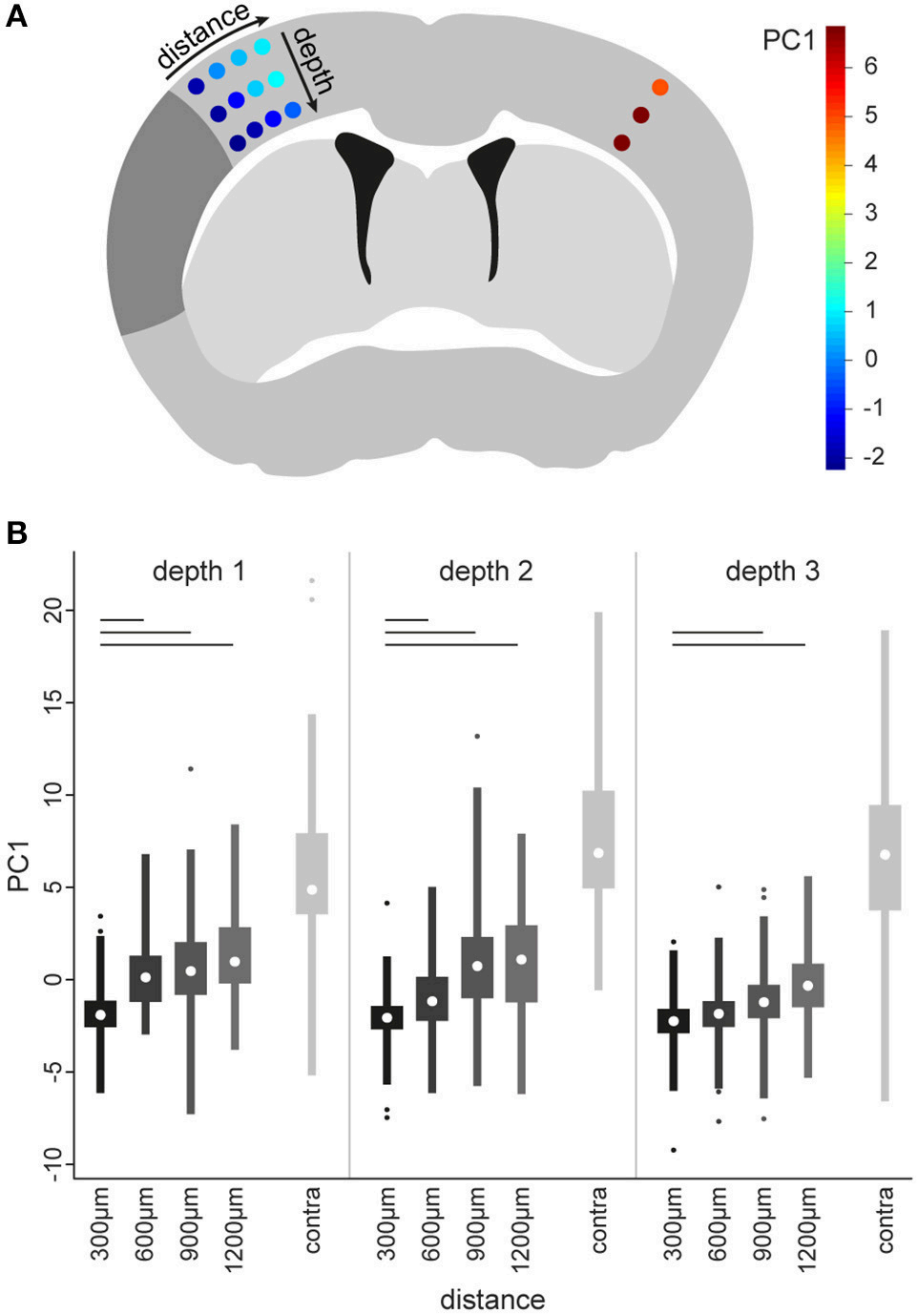
Identification of morphological differences between microglia of the peri-infarct area. To determine microglia morphology in various distances from the infarct area, multiple image stacks were acquired in a grid-like spatial arrangement **(A)**. The grid contained four distances in the ipsilateral cortex (i.e., 300, 600, 900, and 1,200 μm from the infract border) at three different depths (i.e., depth 1, 2, and 3). In addition, a location in the contralateral cortex (contra) homotypic to the 300 μm location was included, at the same 3 depths. The positions of the grid are colored according to the median scores of the first principal component (PC1), calculated as described in the main text for a new set of 5 animals 5 days after stroke (see color bar in **A**). These colors show increasing scores for PC1 with increasing distance from the infarct. Boxplots **(B)** of PC1 scores for all microglia at each imaging position in the grid. A Kruskal–Wallis test was applied to all positions in the grid. Significant *post-hoc* comparisons of ipsilateral positions to the 300 μm position at the same depth are indicated by bars (*p* < 0.001, corrected for multiple comparisons).

We aimed to test the sensitivity of the identified compound score to not only discriminate between surveilling (contralateral) and activated (perilesional) microglia, but also within the activated microglial population. Therefore, we analyzed microglia in a new set of five mice, at three different cortical depths and with increasing distance (300, 600, 900, and 1,200 μm) to the border of the infarct core. The three cortical levels were also assessed in the contralateral hemisphere (Figure 5A). The analysis was then conducted as established in our exploratory and validation study, including PCA and determination of PC1 as a compound score. Using this score, we identified a gradient in microglial morphological changes with increasing distance to the border of the infarct. Differences in microglial shape, as determined by the PC1 score, were significant between the area next to the infarct core and more distant areas at all three cortical levels (Figure 5B). These findings demonstrate that the algorithm is sufficiently robust and sensitive to also detect minor, gradual differences of microglia morphology within the activated microglia population.

### Comparison between manual and automated analysis of microglia morphology

**Figure 6:**
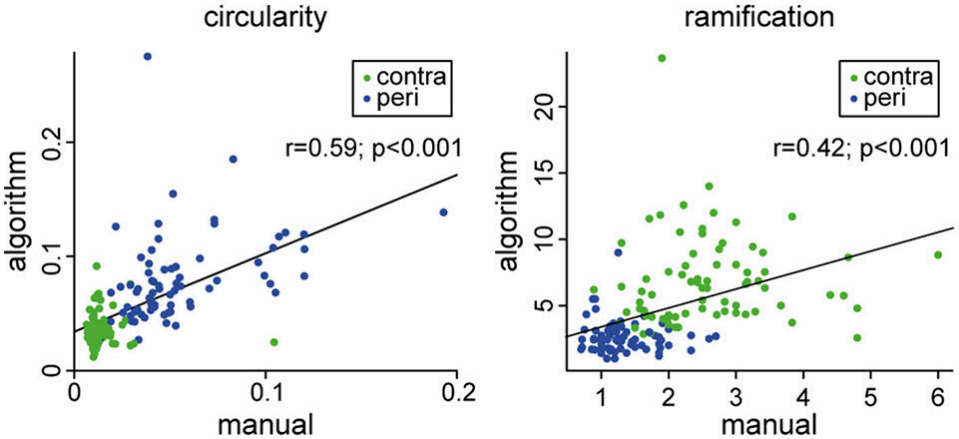
Comparison between human rater (manual) and the automated analysis (algorithm). Circularity was calculated in the same manner for both procedures. Ramification was calculated by the Shoenen ramification index for the manual procedure and as the “end-nodes per branch” feature for the automated procedure.

To test the comparability of results from our automated analysis with results from the human rater, we correlated the scores of the manually and automatically extracted circularity index. In addition, the Shoenen ramification was correlated to the most similar feature from the automated analysis, i.e., “end-nodes-per-branch” (Figure 6). For both morphology markers, we obtained a highly significant positive correlation between manual and automated analysis (Figure 6), indicating a good comparability of results. We further compared the estimated hands-on time for manual and automated analysis. While manual analysis requires ~4–5 min of hands-on analysis time per cell by a trained investigator, the automated algorithm requires only a few minutes of hands on time for file handling and starting the program. Thereby, the manual analysis for example of 100 cells will require ~6–7 h of analysis time by an investigator, in contrast to only ~5 min of hands-on time and the remaining computation being done independent of human input by the automated algorithm.

## Discussion

Microglia are among the first cells to react to brain injury such as a stroke (Streit et al., 2004; Kawabori and Yenari, 2015). A main feature of microglia is their rapid morphological change upon activation, which is characterized by retraction of the numerous, fine processes and gradual acquisition of an amoeboid shape (Anttila et al., 2016). Indeed, *in vivo* two photon and confocal live-imaging in living mice after focal lesion showed immediate retraction of microglial processes and polarization toward the lesion site (Nimmerjahn et al., 2005; Haynes et al., 2006; Davalos and Akassoglou, 2012). Thus, already finest changes in the broad spectrum of microglia morphology are helpful and necessary to give a first impression of microglial activation, since this is usually a continuous process and not divided into distinct shape classes. However, the currently available tools for histological assessment of microglia morphology are still insufficient for assessing changes in the complex cell shape of individual microglial cells. Many of them are prone to rater bias due to the requirement of manual input and are very time consuming (Karperien et al., 2013; Xu et al., 2016).

To overcome these limitations of previously used methods, we created a fully automated algorithm, which analyzes microglia morphology based on fluorescently labeled confocal Z-stacks. The most important advantage of our algorithm is the analysis of microglia morphology completely independent from manual input. Thus, rater-dependent bias can be avoided. In addition, our newly developed tool allows 3D reconstruction of individual microglial cells and characterizing their complex shapes. Besides these improvements to currently available methods, our automated analysis tool is fast and convenient to apply, thus facilitating efficient analysis also of very large cell numbers.

Our method provides a broad panel of features, which can be extracted for analysis of microglia morphology. Among these features, we prove 17 to be highly discriminative between microglia in the peri-infarct and the contralateral cortex, shown by high AUC values in the ROC analysis. However, multiple features would require multiple statistical comparisons entailing corrections for this multiplicity. Alternatively, one single feature could be chosen *a priori*, without knowing whether this feature is also the most informative in a new study. In order to address these issues, we propose to create a compound score using PCA. By including the information of multiple features, this compound score is a potentially more robust marker for quantifying microglia morphology than any one single feature.

We developed the algorithm on an exploratory dataset of 4 mice 3 days after stroke. In a second dataset of additional five mice, which underwent the same treatment procedures, we independently validated our results by reproducing the high discrimination performance for the two locations in the cortex after stroke. Thus, we can largely exclude overfitting of the algorithm to the exploratory dataset. We could further show that the algorithm-based analysis of microglia morphology shows comparable results to commonly used features, which depend on time-consuming and potentially biased manual processing steps.

In general, image-based analysis of microglia morphology has some limitations, which also affects the presented automated pipeline. First, an excellent image quality of the raw data is indispensable for performing the analysis. A critical step is tissue preparation including proper perfusion and tissue fixation in order to achieve artifact-free staining. Therefore, an optimized protocol from tissue preparation until staining of the thick tissue sections is important, which is provided in detail in this report. To assure that only qualitatively appropriate images are included in the analysis, the presented algorithm applies automated quality control, which also helps to avoid manual and possibly biased selection of images. We verified the error rate of our algorithm by manually evaluating the outliers identified in the PCA plot (see Figure 4) as our algorithm allows the back-tracing of individual data points to the respective cell on the image stack. Thereby, we determined the error rate for false cell identification to be < 1% which represents an excellent performance.

The steps in our tissue preparation protocol and quality control cover improvements to previously described methods for microglia morphology analysis. The thickness of analyzed tissue slices is important to allow the analysis of entire microglial cells including their branches. However, this criterion is often not met by previously described methods, where very thin sections (10–30 μm) were used for two-dimensional analysis. This limits the accuracy of describing three-dimensional morphology of microglia (Kozlowski and Weimer, 2012; Plog et al., 2014; Verdonk et al., 2016; Ding et al., 2017). With our optimized protocol for staining and imaging, we are able to analyze the three-dimensional structure of entire microglial cells. Additionally, some of previous methods for microglial shape analysis depend on microglial reporter mouse lines such as the CX3CR1-GFP strain, which might complicate the applicability in models where genetic modified animals are required. We avoid this limitation by staining microglia for Iba1. Thus, the method we present is precisely adapted to reach high quality of microglia Z-stack images, which are the base for accurate analysis by our automated algorithm. Another major limitation of previously presented automated algorithms is the lack of public access to the scripts, which would allow researchers to apply the methods. In contrast, we provide our algorithm together with example data as free and open source software. This allows other researchers to easily examine and apply the code.

Changes in microglia morphology are not to be set equal with the functional properties of a microglial cell. On the one hand, the morphology is a reasonable marker for studying the general activation state of microglia in reaction to a pathogen or tissue injury. On the other hand, microglia have very pleiotropic functions, which become apparent through complex transcription profiles demonstrated in recent publications (Hickman et al., 2013; Crotti and Ransohoff, 2016; Keren-Shaul et al., 2017). Thus, analysis of morphology based on immunohistological sections provides valuable topographical information about microglial activation. However, for investigating microglial function additional methods are necessary, e.g., flow cytometry and transcriptomic studies.

Taken together, we introduce a novel, fully automated, reliable, and objective method to analyze microglia morphology. Due to the high discrimination performance, we expect that the sensitivity of the tool is highly suitable to distinguish morphological changes of microglia in a broad range of brain diseases. The algorithm is a powerful tool to study the effect of novel treatments and experiments on microglial activation in a variety of disease models. Besides stroke, microglia play an essential role in several acute and chronic neurodegenerative diseases, such as Alzheimer's disease, Parkinson's disease, amyotrophic lateral sclerosis and multiple sclerosis (Teismann et al., 2003; Jack et al., 2005; Qian and Flood, 2008; Brites and Vaz, 2014; Doens and Fernández, 2014). In conclusion, our novel algorithm—which is publicly available—adds a valuable tool to the field of studying microglia morphology in various brain disease models.

## Author contributions

SH and BG: performed the experiments and data analysis and wrote the manuscript. BG and MD: developed the automated algorithm and implemented it in Matlab. CB: performed the surgeries for distal middle cerebral artery occlusion. GL: helped establishing the staining protocol. AL and MD: initiated the study, conceived the study design and critically revised the manuscript. All authors read, edited, and approved the manuscript.

### Conflict of interest statement

The authors declare that the research was conducted in the absence of any commercial or financial relationships that could be construed as a potential conflict of interest.
