# Supplementary material for: Automated Morphological Analysis of Microglia After Stroke
Source: Front Cell Neurosci. 2018 Apr 19;12:106. doi: 10.3389/fncel.2018.00106 (PMC5917008; doi:10.3389/fncel.2018.00106)
Supplement: Supplementary file 3 [file DataSheet1.pdf]

Supplementary Material

Automated Morphological Analysis of Microglia After Stroke

S. Heindl[*], B. Gesierich[*], C. Benakis, G. Llovera, M. Düring[#], A. Liesz[#]

Correspondence: PD Dr. Arthur Liesz: arthur.liesz@med.uni-muenchen.de

1 Supplementary Methods

Automated Analysis

Image segmentation

Thresholds for image segmentation were defined by detecting edges in the images using the Sobel method as implemented in MATLAB. This method finds edges using the Sobel approximation to the derivative. A Kernel distribution was then fitted to the edge intensities, and used to define three different intensity thresholds, depending on the probabilities indicated by the Kernel distribution for the edge intensities.

Threshold 1 (ThrMax): intensity having the maximum probability at the edges.

Threshold 2 (ThrLower): intensity below ThrMax with the probability being 2/3 of the maximum probability.

Threshold 3 (ThrUpper): intensity above ThrMax with the probability being 2/3 of the maximum probability.

These thresholds were determined for each image in the Z-stack, resulting in sequences of thresholds, which were then smoothed using a moving average with a moving window including three neighboring images.

The microglia cells and nuclei were then segmented from the background by creating following binary masks and their logical unions ($\cup$) and intersections ($\cap$).

Microglia segmentation ($Mask_{Cell}$):

Mask 1: pixel in anit-Iba1 channel with intensities > ThrUpper

Mask 2: pixel in anit-Iba1 channel with intensities > ThrMax

Mask 3: pixel in DAPI channel with intensities < ThrLower

$Mask_{Cell}$: Mask 1 $\cup$ (Mask 2 $\cap$ Mask 3)

Mask for microglia cell nuclei ($Mask_{Nuclei}$):

Mask 4: pixel in DAPI channel with intensities > ThrMax

Mask 5: pixel in anit-Iba1 channel with intensities > ThrUpper

$Mask_{Nuclei}$: Mask 4 $\cap$ Mask 5

The $Mask_{Cell}$ was refined in several steps:

1. Removal of small clusters with a volume less than 10 μm^3 from $Mask_{Cell}$.

2. Filling of holes in $Mask_{Cell}$. Holes were determined in 3D as connected out-mask voxel cluster without connection to the borders of the 3D volume.

3. Definition of the branches ($Mask_{Branches}$) and soma of the cells ($Mask_{Soma}$). Branches were defined as the parts of the whole cell mask ($Mask_{Cell}$) with a diameter being in any direction less than 3 μm. This was done using the MATLAB function 'imopen' with a spherical structuring element of radius 1.5 μm.

4. Filling of small gaps in $Mask_{Cell}$ in the immediate vicinity of the soma. Such gaps might potentially arise from the usage of different thresholds for the creation of the masks combined to build the $Mask_{Cell}$. This was done by applying the MATLAB function 'imclose' with a spherical structuring element of radius 1.2 μm to the $Mask_{Cell}$, inclusively masking the gap filled $Mask_{Cell}$ by a dilated soma mask and adding the original, non-gap filled $Mask_{Cell}$. The dilation of $Mask_{Soma}$ was done using the MATLAB function 'imdilate' with a spherical structuring element of radius 1.2 μm.

The $Mask_{Nuclei}$ was refined in following way:

1. Masking the $Mask_{Nuclei}$ with the $Mask_{Soma}$ to remove artefacts which might result from overlaps of Mask4 and Mask5 in areas where microglia cell branches lie in very close proximity to non-microglia nuclei.

2. Removal of small clusters with volume less than 10 μm^3 from $Mask_{Nuclei}$.

After these refinement steps, the $Mask_{Soma}$ and $Mask_{Branches}$ were redefined within the $Mask_{Cell}$, in the same way as explained above, but allowing a larger diameter for branches (diameter less than 3.6 μm), thereby restricting the soma to areas within the mask with larger diameters. Voxel cluster in the $Mask_{Soma}$ were removed, if no nucleus was found within its area in the $Mask_{Nuclei}$.

The $Mask_{Soma}$ was dilated ($Mask_{SomaDilated}$) using the MATLAB function 'imdilate' with a spherical structuring element of radius 1.2 μm and by masking it inclusively by the $Mask_{Cell}$.

The purpose of $Mask_{SomaDilated}$ was twofold: i) Include minor bumps on the surface of the soma into the soma mask, instead of considering them as cell branches. ii) Separate cell branches, which share the same basis or are connected by ridges on the surface of the soma.

Definition of the skeleton and segregation of cells

The skeleton resulting from the watershed segmentation was refined, in order to avoid artificial cycles in the skeleton, lying internally of enlarged volumes in the cell soma or branches. This was done in two ways. i) Watershed segments which had no or very little contact to the surface of the cell were merged to the neighboring watershed segment, to which it had the largest area of contact. ii) In regions of the cell where more than two watershed segments met, edges were removed by giving preference to edges between watershed

segments with larger area of contact over those with smaller area of contact until all cycles were broken up.

Major branches of the skeleton were defined from the skeleton nodes and edges being located inside the $Mask_{Branches}$. To define distinct branches, these nodes were split into subsets meeting the requirement that each pair of their nodes is connected by a pathway of nodes and edges being part of the same subset. In addition, these subsets were only considered to be major branches of the cell, if any pair of its nodes was connected by a pathway of edges being at least 2 µm long.

Branch segments were defined as all possible pathways of nodes and edges with exactly two nodes being connected by a number of edges unequal two (i.e. end-/branching-nodes).

Cycles in the branches were defined as pathways of edges and nodes wherein a node is reachable from itself.

Definition of morphological features

Sphericity is a measure of the compactness of a 3-dimensional object, and a function of its volume *V* and surface area *A*. A sphere would be the most compact 3D object having a score of one. Sphericity was calculated as:

$$sphericity = \frac{\pi^{\frac{1}{3}}\,(6V)^{\frac{2}{3}}}{A}$$

Circularity is a measure of the compactness of a 2-dimensional object, and a function of its area *A* and perimeter *P*. A circle would be the most compact 2D object having a score of 1. It was calculated for the 2D projection of the cell mask along the Z-axis:

$$circularity = \frac{4\pi A}{P^2}$$

Solidity was calculated for the 2D projection of the cell mask along the Z-axis as the proportion of the pixels in the convex hull that were also in the cell.

The length of branches was defined in two ways:

1. "branch length skeleton" was defined as the longest of the pathways of edges and nodes between the first node outside the soma and any end-node in the same branch.

2. "branch length air-line" was defined as the Euclidian distance between the same pair of nodes, for which the "branch length skeleton" was defined.

Graph theory based centrality measures were calculated using the MATLAB function 'centrality'. Node centrality can be considered as the importance of a node for the graph (i.e. the mathematical representation of the skeleton), according to certain criterions. The here used centrality measures were the degree of the nodes, their closeness and their betweenness.

2 Supplementary Figures and Tables

2.1 Supplementary Figures

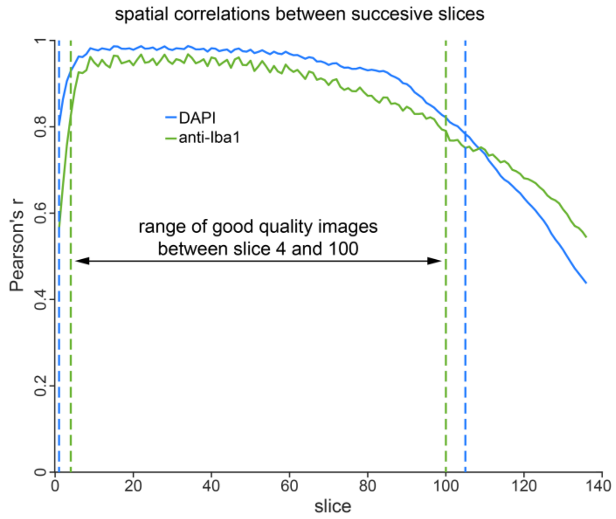

Supplementary figure 1: Assessment of image quality using spatial correlations between successive slices. Spatial correlation between neighboring images (slices) increase with signal strength and decrease with noise, and hence is related to the signal-to-noise ratio. Images were removed from the top and bottom of the image stack, if spatial correlations with their neighboring images were falling below a threshold of 0.78 in either of the two channels (DAPI and anti-Iba1 staining). Dashed lies indicate the range of over-threshold, good quality images in the DAPI (blue) and anti-Iba1 (green) channel, respectively. The overlap of these two ranges (here coinciding with the range of over-threshold images in the anti-Iba1 channel) was used for analysis.

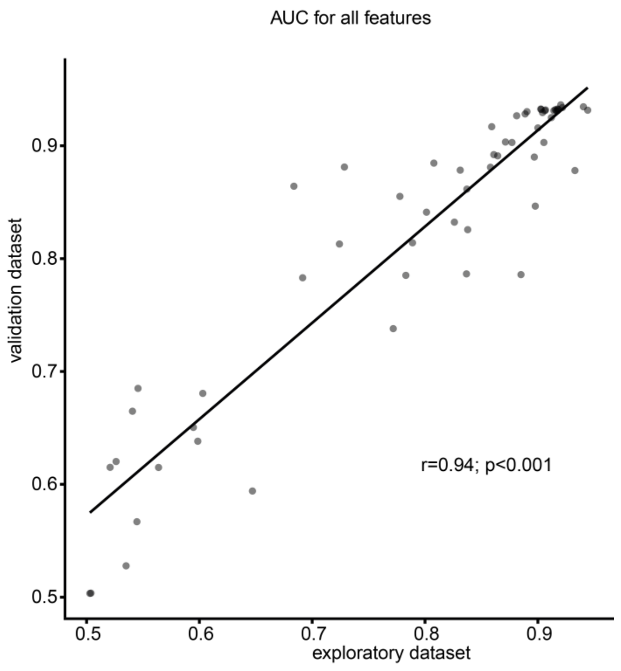

Supplementary figure 2: Validation of the automated analysis. In the validation dataset, each shape feature showed a comparable or even higher performance to discriminate between cells in the peri-infarct area and in the contralateral hemisphere, as estimated by the area under the curve (AUC) from receiver operating characteristic (ROC) analysis. Each data-point represents the AUC scores for one feature, as calculated in the exploratory dataset and in the independent validation dataset.

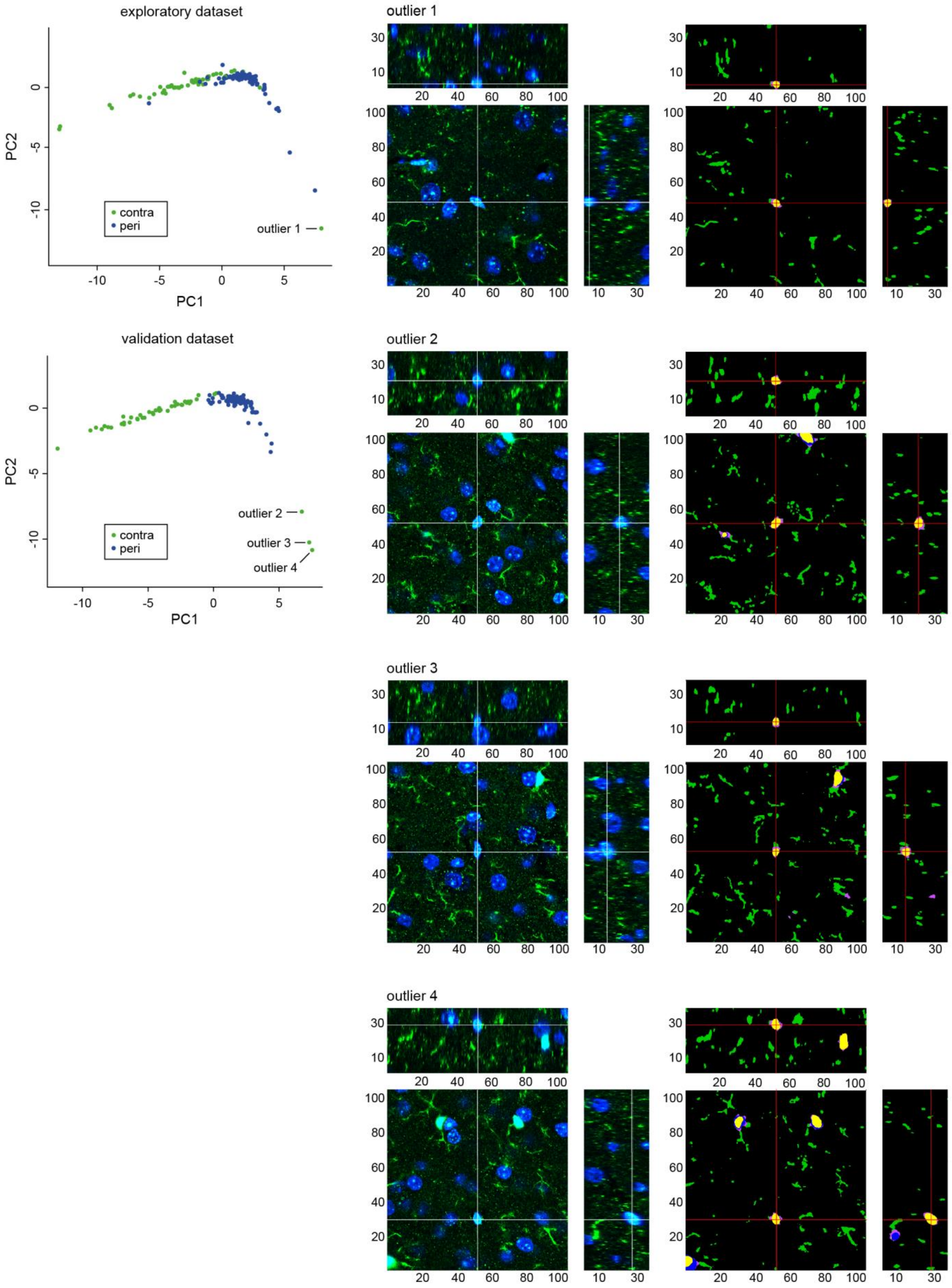

Supplementary figure 3: Identification and control of outliers. Principal component analysis (left column) revealed four outliers along the axes of PC1 and PC2: one cell in the exploratory dataset and three cells in the validation dataset. The individual outliers are visualized by orthogonal slices through the center of the outlier, in a cutout of the raw confocal image stack (middle column) and the segmented cells mask (right column). Intersections of the orthogonal slices are indicated by cross-hairs. Scale is indicated in µm.

Supplementary video 1: Skeleton of cells throughout a whole Z-stack. The video starts with a static picture of the maximum-intensity projection along the Z-direction of the image stack, including anti-Iba1 (green) and DAPI (blue) staining. Subsequently a projection of the segmented microglia cells (green) and nuclei (blue) is shown. The skeleton is overlayed, with skeleton nodes colored according to cell identity (colors chosen at random). The view zooms in, to show a magnified view of a part of the skeleton in rotation.

Supplementary video 2: Surface model and skeleton of a segregated cell in rotation. The initially opaque surface of the cell becomes gradually transparent, in order to show the underlying skeleton model of the cell structure. Skeleton nodes are represented by spheres, with colors indicating their properties. Red: node at the center of the soma; Orange: all other nodes inside the soma; Green: branching points; Pink: end-points of the skeleton; Blue: nodes which were connected to neighboring cells before segregation into individual cells; Brown: all other nodes inside the branches.

2.2 Supplementary Tables

Supplementary Table 1: Shape features

Label, AUC score, type and definition of each of the 59 shape features. For features marked by an asterisk (*) additional explanation is available in the Supplemental Material section "Definition of morphological features". The type of a feature indicates whether it is based on simple shape properties, like volume or surface area (simple); based on skeleton properties (skeleton); or based on graph theoretical measures of centrality (graph). For features which could be defined for each node or branch of the cells skeleton, the scores for 5 percentiles were extracted: the minimum, 25th percentile, median, 75th percentile and maximum. These percentiles are indicated by the suffices P0, P25, P50, P75 and P100 (added to the label of the feature). Features, which were selected for the PCA analysis, are highlighted in light blue.

label	AUC	type	definition
volume soma	0.68	simple	volume of soma
volume branches	0.72	simple	volume of all branches
volume cell	0.56	simple	volume of the cell
surface area cell	0.78	simple	surface area of the cell
sphericity *	0.90	simple	measure of compactness of the cell in 3D
circularity *	0.91	simple	measure of compactness of the cell in 2D
solidity *	0.84	simple	proportion of the pixels in the convex hull that are also in the cell
volume P0	0.78	simple	volume of nodes; minimum
volume P25	0.89	simple	volume of nodes; 25th percentile
volume P50	0.90	simple	volume of nodes; median
volume P75	0.93	simple	volume of nodes; 75th percentile
volume P100	0.73	simple	volume of nodes; maximum
nodes total	0.89	skeleton	total number of nodes in the skeleton
branching nodes	0.90	skeleton	number of branching nodes (degree > 2)
end-nodes	0.86	skeleton	number of blind ending nodes (degree = 1)

branching nodes ratio	0.84	skeleton	ratio branching nodes/nodes total
end-nodes ratio	0.80	skeleton	ratio end-nodes/nodes total
branches	0.65	skeleton	number of major branches
nodes in branches	0.90	skeleton	number of nodes located in the branches
nodes per branch	0.94	skeleton	nodes per major branch
end-nodes in branches	0.89	skeleton	number of nodes located in the branches, having degree 1 (i.e. branch end-nodes)
end-nodes per branch	0.91	skeleton	end-nodes per major branch
branch segments	0.91	skeleton	number of branch segments
nodes per branch segment	0.77	skeleton	nodes per branch segment
segments per branch	0.94	skeleton	segments per major branch
branch cycles	0.91	skeleton	number of cycles in the branches
branch length skeleton P0 *	0.60	skeleton	length of major branches, following the edges of skeleton; minimum
branch length skeleton P25	0.79	skeleton	length of major branches, following the edges of the skeleton; 25th percentile
branch length skeleton P50	0.87	skeleton	length of major branches, following the edges of the skeleton; median
branch length skeleton P75	0.90	skeleton	length of major branches, following the edges of the skeleton; 75th percentile
branch length skeleton P100	0.86	skeleton	length of major branches, following the edges of the skeleton; maximum
branch length air-line P0 *	0.59	skeleton	length of the air-line connecting starting/end nodes of major branches; minimum
branch length air-line P25	0.69	skeleton	length of the air-line connecting starting/end nodes of major branches; 25th percentile
branch length air-line P50	0.83	skeleton	length of the air-line connecting starting/end nodes of major branches; median
branch length air-line P75	0.86	skeleton	length of the air-line connecting starting/end nodes of major branches; 75th percentile
branch length air-line P100	0.81	skeleton	length of the air-line connecting starting/end nodes of major branches; maximum
branch sinuosity P0	0.60	skeleton	ratio branch length skeleton/branch length air-line; minimum
branch sinuosity P25	0.84	skeleton	ratio branch length skeleton/branch length air-line; 25th percentile
branch sinuosity P50	0.90	skeleton	ratio branch length skeleton/branch length air-line; median
branch sinuosity P75	0.86	skeleton	ratio branch length skeleton/branch length air-line; 75th percentile

branch sinuosity P100	0.83	skeleton	ratio branch length skeleton/branch length air-line; maximum
degree cell	0.53	graph	number of neighboring cells to which the cell is connected by touching branches
degree cell multiple	0.52	graph	number of connections to neighboring cells, counting multiple connections to the same cell
degree soma *	0.55	graph	number of edges connected to the node in the center of the soma
degree P25	0.54	graph	number of edges connected to each node; 25th percentile
degree P50	0.50	graph	number of edges connected to each node; median
degree P75	0.50	graph	number of edges connected to each node; 75th percentile
degree P100	0.54	graph	number of edges connected to each node; maximum
closeness soma *	0.92	graph	closeness of the node in the center of the soma
closeness P0	0.91	graph	closeness of nodes; minimum
closeness P25	0.92	graph	closeness of nodes; 25th percentile
closeness P50	0.92	graph	closeness of nodes; median
closeness P75	0.92	graph	closeness of nodes; 75th percentile
closeness P100	0.92	graph	closeness of nodes; maximum
betweenness soma *	0.88	graph	betweenness of the node in the center of the soma
betweenness P25	0.54	graph	betweenness of nodes; 25th percentile
betweenness P50	0.92	graph	betweenness of nodes; median
betweenness P75	0.92	graph	betweenness of nodes; 75th percentile
betweenness P100	0.88	graph	betweenness of nodes; maximum
